# Dose Modulation Strategies in Psoriatic Patients: Real‐Life Pilot Comparison Between Risankizumab and Guselkumab up to 12 Months After Dose Spacing

**DOI:** 10.1111/exd.70062

**Published:** 2025-02-16

**Authors:** Luca Mastorino, Paolo Dapavo, Michela Ortoncelli, Eleonora Bongiovanni, Yingying Liao, Francesco Leo, Pietro Quaglino, Simone Ribero

**Affiliations:** ^1^ Dermatologic Clinic, Departement of Clinical Medicine University of Turin Turin Italy

**Keywords:** biologics, de‐escalation, dose spacing, IL‐17 inhibitors, IL‐23 inhibitors, psoriasis

## Abstract

The possibility of modulating the treatment regimen regarding dose reduction (de‐escalation) or dose augmentation (escalation) in psoriasis biological treatment has been of increasing interest. De‐escalation strategies include reducing the single therapeutic dose, the mg/kg ratio or the number of injections, or dose‐spacing (D‐S), that is, extending the interval between administrations. Data regarding dose de‐escalation, in particular D‐S, on IL‐23, are lacking to date. The present pilot study is a cohort study with a retrospective analysis of the general characteristics and effectiveness outcomes of psoriatic patients undergoing therapeutic biologic D‐S of risankizumab and guselkumab. Ninety‐four patients, 32 (34.04%) treated with guselkumab and 62 (65.96%) treated with risankizumab, underwent dose modulation by D‐S of 50% of the approved range. The mean PASI decreased from 12.15 (5.43 SD) to 0.15 (0.46 SD) at D‐S time. Attainment of PASI100 was rapid: 88.3% at the D‐S date, remaining stable over the following year, reaching 100% of patients observed 12 months after D‐S. Similar is the trend for PASI 90 and PASI <=1 with 91.49% and 97.87% of achievement at D‐S date, and all patients observed at 12 months post‐D‐S. The 12‐month drug survival of the D‐S regimen was 89.4%. Guselkumab showed a D‐S drug survival of 93.3% versus 89.5% of risankizumab. No differences in mean PASIs at each time point were found between guselkumab and risankizumab. To conclude therapeutic modulation of IL‐23 inhibitors in psoriatic patients who have achieved response stability seems a legitimate therapeutic strategy to maintain efficacy and safety.

## 1 Introduction

Psoriasis is an inflammatory skin disease characterised by erythematous scaly plaques or patches involving extensor and palmoplantar surfaces of the body, scalp, and nails [1]. Psoriasis afflicts 125 million people with a prevalence of 0.5% in Asia and 2.5%–8% in Europe [2].

Mild form of psoriasis can be treated with topical treatments such as topical corticosteroids, vitamin D analogs, steroids plus vitamin D analog combinations, calcineurin inhibitors, and keratolytics [2]. Phototherapy (narrow‐band UVB or PUVA) is an option for moderate forms of psoriasis, and systemic therapies such as methotrexate, cyclosporin, and acitretin can be considered for moderate‐to‐severe forms of psoriasis as first‐line treatments [2].

Biologics, such as IL‐23 inhibitors have demonstrated high efficacy and safety in the treatment of moderate–to‐severe psoriasis in recent years [3]. These therapies allow the achievement of Psoriasis Area Severity Index (PASI) 90 and 100, having an important impact on quality of life such as the Dermatology Life Quality Index (DLQI) 0/1 [3]. The possibility of modulating the treatment regimen in terms of dose reduction (de‐escalation) or dose augmentation (escalation) has been of increasing interest [4].

De‐escalation strategies consist in reducing the single therapeutic dose, reducing the mg/kg ratio or the number of injections or dose‐spacing (D‐S), that is, extending the interval between administrations [4]. Several real‐life experience on dose modulation are available for dupilumab in severe atopic dermatits [5, 6], and rheumatological diseases [7], data regarding dose de‐escalation, in particular D‐S, on IL‐23 are lacking to date [8, 9].

De‐escalation in selected patients is a cost‐saving strategy in countries with universalistic national services [4]. The reduction of injections over the course of the year could reduce the psychological burden in psoriasis patients, increase compliance, and credibly lead to a reduction in possible adverse events [4]. Although there are no universally agreed‐upon indications in the selection of patients who are candidates for D‐S, it is the opinion of the authors that the ideal patient who is a candidate for this therapeutic strategy is a patient with no joint involvement, and with a stabilised PASI 90/100 attainment over at least 6 months. In our clinic, we obtained the systemic off‐label authorization for the de‐escalation of guselkumab and risankizumab among IL‐23 inhibitors in psoriatic patients showing a good and stable response at these treatments. In the present study, we aim to analysed effectiveness, safety of de‐escalation strategy of these two biologics.

## 2 Methods

The present pilot study is a cohort study with retrospective analysis of the general characteristics and efficacy outcomes of psoriatic patients undergoing therapeutic biologic D‐S of Risankizumab and guselkumab. All patients older than 18 years, undergoing a D‐S of 50% of the normal range of the two biological drugs followed at the Dermatology Clinic of the University of Turin from January 2017 to December 2022, were enrolled.

Primary Objectives:

- Descriptive analysis in observed cases of baseline characteristics, super responders status, and trends in mean PASI, PASI100, PASI90, and PASI <=1 at baseline (date of biological start) at 16 weeks, at 28 weeks, at the time of dose‐spacing, and at 3, 6, 9 and 12 months after dose‐spacing.

Secondary Objectives:

- Drug survival analysis of dose‐spaced regimen and of treatments' use.
- Comparative analysis between risankizumab and guselkumab for baseline characteristics, super responders status, mean PASI, PASI100, PASI90, and PASI <=1, at 16 weeks, 28 weeks, D‐S date and time points following D‐S at 3, 6, 9 and 12 months.
- Safety analysis: every adverse event reported in the records at each time point from treatment start, to last follow‐up was highlighted.

PASI <=1 was selected as an outcome by the authors to assess the maintenance of minimal disease activity in patients who entered D‐S with generally low PASIs of less than 3; more widely accepted absolute outcomes such as PASI <=2/3 would not adequately read maintenance of response.

### 2.1 Approved Drug Dose Regiment

Traditional approved regimen for considered biological agents after initial specific induction (please refer to specific datasheet):

- Guselkumab 100 mg sc inj every 8 weeks
- Risankizumab 75 mg 2 sc inj every 12 weeks (150 mg fl not available at the moment of the study)

De‐escalated regimen analysed:

- Guselkumab 100 mg sc injection (inj) every 12 weeks
- Risankizumab 75 mg 2 sc inj/150 mg 1 sc inj every 18 weeks

Super responders (SRs) definition:

- SRs according to GUIDE study [10]: PASI 100 achievement at 20 and/or 28 weeks of treatment (SRs GUIDE).
- SRs according to authors' definition (AD) [11]: PASI 100 achievement at 20 and 28 weeks of treatment.

### 2.2 Statistical Analysis

Continuous variables were described by mean ± SD, based on the distribution of each variable. For categorical variables, absolute and relative frequencies were provided. Percentages were based on the number of non‐missing values. Linear regression (T Student's for continuous variable and chi‐square test for categorical variables) for comparative analysis. To analyse patients' D‐S discontinuation of modulated dose regimens, survival analysis techniques were employed. The event was defined as dose‐escalation discontinuation for any reason while the time of observation was calculated from the date of D‐S to the last follow‐up visit.

Statistical analysis was conducted with STATA 15.1 SE (StataCorp., 2017); all tests were two‐sided, and the statistical significance was set to *α* = 0.05.

The present study was approved by our Institutional Review Board under protocol SS‐Dermo‐20.

## 3 Results

Ninety‐four patients, 32 (34.04%) treated with guselkumab and 62 (65.96%) treated with risankizumab, underwent dose modulation by D‐S of 50% of the approved range. The mean age was 53.41 years (16.47 SD), and 59.67% of patients were male, with no significant differences between the 2 drugs. Baseline characteristics of the overall population and individual treatments are summarised in Table 1. Patients treated with risankizumab had a higher mean BMI (body mass index), although not significantly so: 26.8 (5.06 SD) versus 23.22 (3.07 SD), *p* = 0.056. Patients treated with guselkumab were largely naive to biological drugs, 93.8% versus 62.3% of those treated with risankizumab. Considering both SRs definitions, patients treated with risankizumab revealed a faster response than patients treated with guselkumab: SRs (GUIDE) 69.4% versus 43.8%, *p* = 0.016; SRs (AD) 58.1% versus 21.9%, *p* = 0.001 (Table 1).

**TABLE 1:** Baseline patient's characteristics.

	Total	Guselkumab	Risankizumab	*p*
Patients N°/%	94/100%	32/34.04%	62/65.96%	
Mean age (SD)	53.41 (16.47)	51.19 (17.41)	54.57 (15.98)	0.349
Sex M N°/%	56/59.67%	17 (53.1%)	39 (62.9%)	0.36
Mean BMI (SD)	25.85 (4.84)	23.22 (3.07)	26.8 (5.06)	0.056
Mean age of onset (SD)	33.47 (18.05)	32.81 (19.26)	33.83 (17.62)	0.837
SRs GUIDE N°/%	57/60.64%	14 (43.8%)	43 (69.4%)	0.016
SRs (AD) N°/%	43/45.74%	7 (21.9%)	36 (58.1%)	0.001
Difficult site N°/% (1 missing)	68/73.1%	22 (71%)	46 (74.2%)	0.741
PsA N°/% (1 missing)	5/5.4%	0 (0%)	5 (8.1%)	0.165
Obesity N°/%	8/8.5%	0 (0%)	8 (12.9%)	0.168
Naïve N°/%	70/74%	30 (93.8%)	38 (62.3%)	0.001
FU before D‐S Mean (SD)	25.2 (10.12)	24.15 (11.87)	25.24 (9.16)	0.824
D‐S interruption N/%	6/6.4%	2 (6.3%)	4 (6.5%)	0.97
Drug interruption N°/%	2/2.13%	1 (3.1%)	1 (1.6%)	0.63

Abbreviations: AD, authors' definition; BMI, body mass index; D‐S, dose spacing; FU, follow‐up; GUIDE, GUIDE definition; N, number; PsA, psoriatic arthritis; SD, standard deviation; SRs, super responders.

The mean time before D‐S was 25.2 months (10.12 SD), superimposed between the 2 treatments: 24.15 (11.87 SD) for guselkumab and 25.24 (9.16 SD) for risankizumab (*p* = 0.824). The mean follow‐up under the D‐S regimen was 7.12 (4.66 SD) months.

**FIGURE 1:**
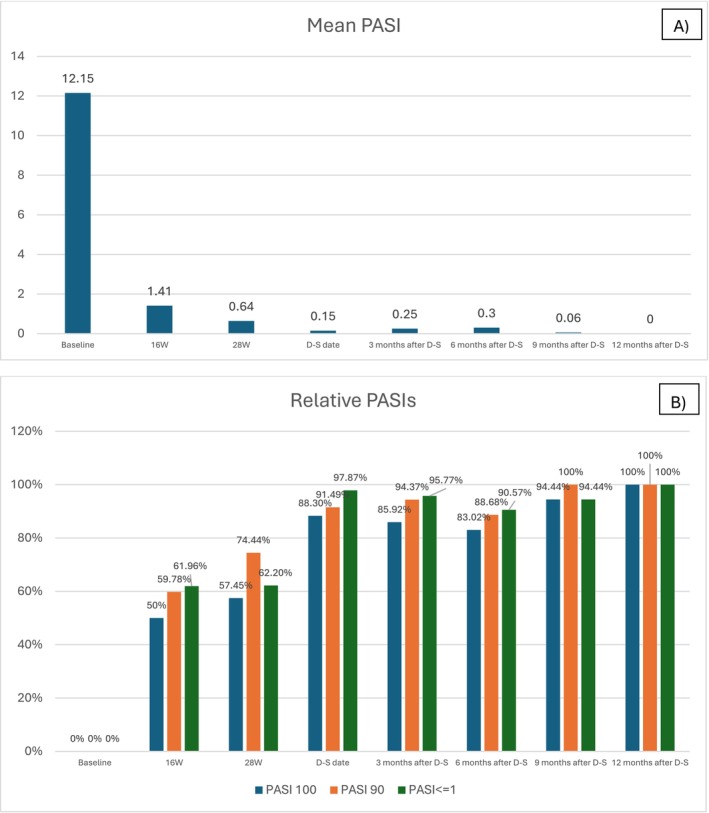
(A and B) Mean PASI, PASI100, PASI90 and PASI = 1 trend from 16 W to 12 months afters D‐S. D‐S, dose spacing; PASI, psoriasis area severity index; W, week.

**FIGURE 2:**
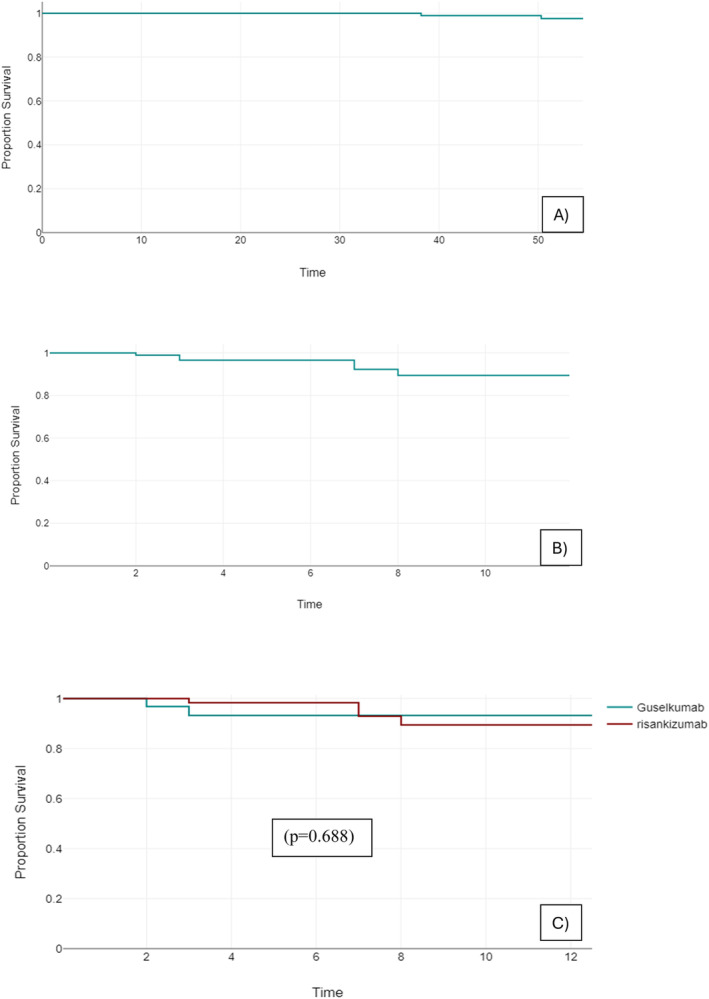
(A) Overall drug survival on patients at risk for both treatments. (B) Overall drug survival of D‐S regimen on patients at risk for both treatments. (C) Comparison of drug survival of D‐S regiment for risankizumab versus guselkumab on patients at risk. On axis time in months, *p*, *p*‐value.

The mean PASI in the population decreased overall from 12.15 (5.43 SD) to 1.41 (1.97 SD) at 16 weeks, at D‐S the mean PASI was 0.15 (0.46 SD), fluctuating from 0.3 to 0 in the year following treatment extension (Table 2, Figure 1A). Attainment of PASI100 was rapid for both cohorts: 57.5% at 28 weeks, 88.3% at the D‐S date, remaining stable over the following year, reaching 100% of patients observed 12 months after D‐S (Table 2, Figure 1B). Superimposable is the trend for PASI 90 and PASI <=1 with 74.44% and 62.2% of patients achieving outcome at 28 weeks, 91.49%, and 97.87% at D‐S date, and all patients observed at 12 months post D‐S (Table 2, Figure 1C,D). Only 2 patients discontinued treatment permanently (2.13%) (both for side effects): 4‐year survival has been 97.6% for both treatments, 1 patient on guselkumab discontinued for migraine, and 1 on risankizumab for arthralgia; no other patients reported adverse events (Figure 2A). The 12‐month drug survival of the D‐S regimen was 89.4% including the two definitive discontinuations and six patients who returned to the standard regimen due to loss of efficacy (Figure 2B).

**TABLE 2:** Mean PASI, PASI100, PASI90 and PASI = 1 trend from 16 w to 12 months afters D‐S.

	Baseline	16 w	28 w	Dose spacing date	3‐months after	6 months after	9 months after	12 months after
Mean PASI (SD)	12.15 (5.43)	1.41 (1.97)	0.64 (0.92)	0.15 (0.46)	0.25 (0.86)	0.3 (0.75)	0.06 (0.24)	0 (0)
Guselkumab	11.1 (4.1)	1.47 (1.27)	0.84 (0.77)	0.25 (0.44)	0.17 (0.39)	0.29 (0.77)	0 (0)	0 (0)
Risankizumab	12.68 (5.96)	1.38 (2.26)	0.53 (0.98)	0.1 (0.47)	0.29 (1.01)	0.31 (0.75)	0.13 (0.35)	0 (0)
*p*‐value	0.188	0.844	0.126	0.129	0.802	0.985	0.696	1
PASI 100%	0%	50%	57.5%	88.3%	85.92%	83.02%	94.44%	100%
Guselkumab	0%	28%	38%	75%	83%	82%	100%	100%
Risankizumab	0%	63%	78%	95%	88%	83%	88%	100%
*p*‐value		**0.001**	**0.001**	**0.004**	0.579	0.929	0.25	1
PASI 90%	0%	58.51%	74.44%	91.49%	94.37%	88.68%	100%	100%
Guselkumab	0%	47%	56%	84%	96%	88%	100%	100%
Risankizumab	0%	67%	84%	95%	94%	89%	100%	100%
*p*‐value		0.065	**0.003**	0.076	0.745	0.944	1	1
PASI <=1%	0%	60.64%	62.2%	97.87%	95.77%	90.57%	94.44%	100%
Guselkumab	0%	50%	41%	100%	100%	94%	100%	100%
Risankizumab	0%	68%	74%	97%	94%	89%	88%	100%
*p*‐value		0.085	**0.002**	0.304	0.546	1	0.25	1

*Note:* Bold values indicate statistical significance (*p* < 0.05).

Abbreviations: D‐S, dose spacing; PASI, psoriasis area severity index; w, week.

**FIGURE 3:**
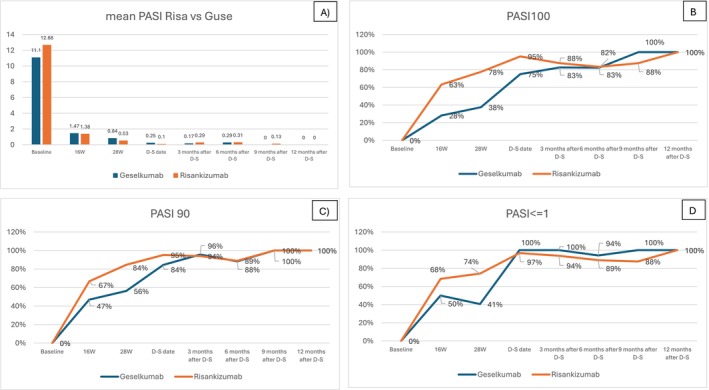
(A) Mean PASI comparison between risankizumab and guselkumab from baseline to 12 months after D‐S. (B) PASI100 comparison between risankizumab and guselkumab from baseline to 12 months after D‐S. (C) PASI90 comparison between risankizumab and guselkumab from baseline to 12 months after D‐S. (D) PAS <=1 comparison between risankizumab and guselkumab from baseline to 12 months after D‐S. D‐S, dose spacing; PASI, psoriasis area severity index; W, week.

Guselkumab showed a drug survival of D‐S regimen of 93.3%, with two patients returned to standard dosing, versus 89.5% of risankizumab, with four patients returned to standard dosing (*p* = 0.688) (Figure 2C). No differences in mean PASIs at each time point were found between guselkumab and risankizumab (Table 2, Figure 3A). But the latter provided a faster response considering PASI 100 with 63%, 78%, and 95% at 16, 28 weeks, and D‐S date, respectively, versus 28%, 38%, and 75% at the same time points as guselkumab (*p* = 0.001 at 16 and 28 weeks, and 0.004 at D‐S date) (Table 2, Figure 3B). The superiority of risankizumab was also observed at 28 weeks considering PASI90 and PASI <=1 (Table 2, Figure 3C,D). The two drugs following D‐S maintained an overlapping achievement of outcomes for patients observed up to 12 months (Table 2, Figure 3).

## 4 Discussion

Recent studies postulate that seeking the minimal necessary doses using an optimised off‐label dose‐reduced regimen might improve the risk‐benefit/ratio and maximise clinical outcomes while reducing the costs of biologic treatment [5].

Therapeutic de‐escalation of guselkumab and risankizumab proved effective and safe in controlling the response obtained at conventional dosing in patients with moderate–to‐severe psoriasis. Over the year following prolongation, a minimal overall worsening of outcomes was observed between 6 and 9 months; however, only 10 patients returned to standard dosing, with an estimated survival of almost 90% at 1 year. Only six patients discontinued D‐S due to loss of efficacy, and two patients due to adverse events. No significant differences were observed between the 2 drugs at D‐S in achieving therapeutic goals. Patients treated with risankizumab showed a faster response in the first weeks of treatment, but at the D‐S date, the two treatments were equal. The D‐S patient population showed similar baseline characteristics to the generally treated psoriatic population, except for a lower rate of patients with psoriatic arthritis (5%) and obesity (6%). These two populations are also subject to lower therapeutic response, as evidenced by real‐life studies, and therefore less likely to be candidates for negative treatment modulation [12]. Guselkumab also showed a bio‐naive patient rate of over 90% compared to 68% for risankizumab.

Regarding safety, D‐S does not seem to be of particular concern, excluding disease recurrence, in the patients observed only two reported an episode of migraine and one episode of arthralgia, respectively. In both cases, the event led to definitive discontinuation of treatment.

In the literature, most of the real‐life studies focus on the modulation of anti‐TNF alpha (adalimumab, etanercept, infliximab), data on secukinumab and brodalumab can only be retrieved from phase II/III studies [8, 12]. The selection of the psoriatic patient candidate for therapeutic de‐escalation is not homogeneous among the various works [13].

Maintenance of these outcomes ranged from 3 months to more than a year before D‐S was experienced [7]. The average savings reported in some papers range from 13% to 19% annually [8].

A Belgian real‐life experience with IL‐17 inhibitors a total of 18.7% of patients (*n* = 25) underwent therapeutic modulation: 16 escalated and 9 de‐escalated (6.7%). Of the 9 de‐escalated patients 2 returned to a conventional regimen [14]. This work proposed therapeutic drug monitoring using blood sample analysis, to select patients eligible to dose de‐escalation. In the case of a patient with a level above the range of normality of biological drugs in the blood, concomitant with a persistent therapeutic response, de‐escalation could be an appropriate strategy [15].

Data available on IL‐23 inhibitors remains scarce, in particular in real‐life setting. GUIDE study demonstrated that guselkumab dosing every 16 weeks was non‐inferior to the standard every 8 weeks dosing interval for maintenance of disease control at W68 in super‐responders patients (PASI 100 at 20 or 28 weeks), assuming ad disease control PASI >=3 [16].

The retrospective cohort study Herranz‐Pinto et al. included a total of 69 patients, of which 45 underwent an ‘on‐demand’ drug reduction strategy of guselkumab. After an initial complete response, patients re‐administered guselkumab only when their absolute PASI reached ≥ 1. The follow‐up was 88 weeks. Patients were divided into four groups: one standard dose group and three groups based on the % DR of the standard dose: one group underwent a reduction of 29% (*N* = 24), the other two of 52% (*N* = 10) and 71% (*N* = 11). All de‐escalated groups showed a significant decrease in PASI between weeks 11 and 20 compared to the baseline. After 1 year, drug survival curves showed a survival rate of 93.5% in the overall population (including patients on standard dose), 94.4% in the group with 29% reduction, and 100% in the other two groups without significant differences between groups (*p* = 0.48) [17].

Gisondi *et al.* proposed an on‐demand treatment for risankizumab in 64 patients who reported a complete response after the first 3 injections (induction phase); the patients resumed the drug only at the onset of a PASI > 1. The mean time between 3 and 14 injections was 32 weeks, compared to 12 with the conventional regimen, rising to 34 and 39 at the next 2 injections [9]. As suggestive as it is, on‐demand therapy has strong limitations, in terms of replicability from a scientific point of view and of increased risk of loss at patient follow‐up, also considering the difficulty of access to treatment centers. A patient with a complete response might lose awareness of the disease and fail to show up for follow‐up; conversely, a patient with severe disease reactivation might be switched to another treatment, failing to access the center treating him. For this reason, we consider it necessary to standardise de‐escalation and possibly proceed to progressively higher D‐S.

Dose modulation strategies using IL‐23 inhibitors are rationally supported by the known effect these specific inhibitors have on so‐called memory T cells [18]. Inhibition of IL‐23 may provide better long‐term results due to its impact on the Treg to CD8+ TRM ratio than inhibition of IL‐17. IL‐23 may contribute to inflammation persisting even after treatment [18]. The differences found between guselkumab and risankizumab are to be found in the small sample size observed at extreme time points, which is more affected by random events such as side effects or temporary loss of response. Furthermore, the characteristics at baseline of patients treated with risankizumab appear more challenging than with guselkumab, having a higher mean initial PASI, more obese, higher mean BMI, and more consistent joint involvement.

The limitations of our study are those intrinsic to real‐life studies, the retrospective condition, the analysis limited to observed cases, the low sample size especially at extreme time points, and the lack of a control group. Moreover, real‐life retrospective analysis fails to mitigate observation bias and selection bias. Specifically in our study, the small sample size makes it impossible to assess the possible condensing effect of concomitant drugs, comorbidities, and possible changes in disease severity during treatment. The absence of a control group in a conventional regimen makes it impossible to define D‐S as a more effective treatment than conventional, however good response stability is observed.

However, our study is one of the first focusing on a dose‐spacing strategy systematically applied to patients treated with an IL‐23 inhibitor.

Randomised, controlled studies such as BeNeBio on all IL‐23 and IL‐17 inhibitors may soon confirm our results [19].

## 5 Conclusions

Therapeutic modulation of IL‐23 inhibitors in psoriatic patients who have achieved response stability seems maintaining efficacy and safety in our cohort; future observations and case–control studies could validate our findings. D‐S could be useful in reducing health care costs, and the therapeutic burden suffered by the patient. No significant differences in effectiveness were found between risankizumab and guselkumab undergoing D‐S. The obese population and patients with joint involvement appear under‐represented, possibly due to greater difficulty in achieving initial response. Further real‐life and randomised studies are needed to better highlight the merits and shortcomings of this therapeutic approach.

## Author Contributions

L.M., wrote the paper, performed the research, designed the research study, and analysed the data. F.L., Y.L., E.B., P.D. and M.O. performed the research. P.Q. contributed essential reagents tools. P.D. contributed essential reagents tools and performed the research. S.R. designed the research study, analysed the data and contributed essential reagents tools. All authors contributed substantially to the work and read and approved the final version of the manuscript.

## Ethics Statement

Ethical approval was obtained from the Institutional Review Board (SS‐Dermo‐20 protocol).

## Consent

All patients written informed consent.

## Conflicts of Interest

The authors declare no conflicts of interest.

## Data Availability

Data available upon reasonable request.
